# Supplementary material for: Stability and Safety Traits of Novel Cyclosporine A and Tacrolimus Ophthalmic Galenic Formulations Involved in Vernal Keratoconjunctivitis Treatment by a High-Resolution Mass Spectrometry Approach
Source: Pharmaceutics. 2020 Apr 20;12(4):378. doi: 10.3390/pharmaceutics12040378 (PMC7238033; doi:10.3390/pharmaceutics12040378)
Supplement: Supplementary file 1 [file pharmaceutics-12-00378-s001.pdf]

pharmaceutics

Supplementary Materials:

Stability and Safety Traits of Novel Cyclosporine A and Tacrolimus Ophthalmic Galenic Formulations Involved in Vernal Keratoconjunctivitis Treatment by a High-Resolution Mass Spectrometry Approach

Daniele Giovanni Ghiglioni [1], Piera Anna Martino [2], Gaia Bruschi [3,*], Davide Vitali [4], Silvia Osnaghi [1], Maria Grazia Corti [1] and Giangiacomo Beretta [5]

1 Fondazione IRCCS Ca' Granda Ospedale Maggiore Policlinico di Milano; Via Francesco Sforza, 28, 20122 Milan (MI), Italy; daniele.ghiglioni@policlinico.mi.it (D.G.G.); silvia.osnaghi@policlinico.mi.it (S.O.); mariagrazia.corti@policlinico.mi.it (M.G.C.)
2 Department of Veterinary Medicine, Università degli Studi di Milano, Via Celoria 10, 20133 Milan (MI), Italy; piera.martino@unimi.it
3 Department of Clinical and Community Sciences, University of Milan, Via della Commenda, 19, 20122 Milan (MI), Italy
4 Bruttomesso pharmacy, galenic laboratory; Piazza Guglielmo Marconi, 20, 26013 Crema (CR), Italy; lab@farmaciabruttomesso.it
5 Department of Environmental Science and Policy, Università degli Studi di Milano, Via Mangiagalli 25, 20133 Milan (MI), Italy; giangiacomo.beretta@unimi.it
* Correspondence: gaia.bruschi@unimi.it

Received: 29 February 2020; Accepted: 16 April 2020; Published: 20 April 2020

Table S1. Research studies collection on Cyclosporine A and Tacrolimus based ophthalmic formulations for Vernal keratoconjunctivitis treatments.

Author	years	Active compound concentration	Formulation type	Pathology	Treatment/experiment duration	Results
			**Cyclosporine based formulations**			
			**Clinical trial**			
BenEzra	1986	2%	Lacrimart	VKC	6 weeks	Signs and symptoms improvement after the first week of treatment.
Secchi	1990	2%	oil	VKC	4–9 months	Within the first 15 days, both symptoms and signs of the condition improved significantly.
Pucci	2002	2%	oil	VKC	4 times a day for 4 months	Signs and symptoms improvement after two weeks of treatment.
Akpek	2004	0.05%	Lacrimart	AKC	4–6 times a day for 1 month	Signs and symptoms improvement after one month of treatment.
Kilic	2006	2%	Lacrimart	VKC	5 times a day for 14 weeks	Signs and symptoms improvement after two weeks of treatment.
Spadavecchia	2006	1% and 1.25%	Lacrimart	VKC	4 times a day for 4 months	Signs and symptoms improvement after two weeks of treatment in both groups.
Oczan	2007	0.05%	Lacrimart	VKC, AKC	2–4 times a day for 6 months	Signs and symptoms improvement after six months of treatment.
Pucci	2010	1–2%	Lacrimart	VKC	2–4 times a day for 2–7 years	Signs improvement during the study

						period.
Lambiase	2011	0.05% and 0.025%	oil	VKC	2 times a day for 6 months	Signs and symptoms improvement during the study period.
Takamura	2011	0.1%	Lacrimart	VKC	6 months	Signs and symptoms improvement after six months of treatment.
Keklicki	2014	0.05%	Lacrimart	VKC	4 times a day for 4 weeks	Signs and symptoms improvement after one month of treatment.
Yucel	2016	0.05%	Lacrimart	VKC	4 times a day for 3 months	Signs and symptoms improvement after one month of treatment.
Leonardi	2019	0.1%	Cationic emulsion	VKC	2–4 times a day for 4 months	Signs and symptoms improvement after four months of treatment.
**Compositional/stability studies**						
Chennell	2017	1–2%	Micellar solution	In vitro	-	The micellar formulation can be stored at 5 °C or at ≤25 °C for up to 6months.
Lallemand	2017	variable	Lacrimart, oil	VKC, dry eye	variable	Restasis and Ikervis are the two major and successful emulsion products
Shen	2018	variable	mPEG-PLA polymer micelles	Human corneal epithelial and rabbits.	-	mPEG-PLA diblock polymer can be potentially used as a nanoscopic carrier to deliver hydrophobic drugs to the ocular region
**Tacrolimus based formulations**						
**Clinical trials**						
Vichyanond	2004	0.1%	ointment	VKC	1–2 times a day for 1 month	Signs and symptoms improvement after one month of treatment.
Miyazaki	2008	0.02%	ointment	VKC, AKC	1–4 times a day for 26 months	Signs improvement during the study period.
Ohashi	2010	0.1%	Lacrimart	VKC, AKC	2 times a day for 1 month	Signs and symptoms improvement after one month of treatment.
Kheirkhah	2011	0.005%	Lacrimart	VKC	4 times a day for 10 months	Signs and symptoms improvement during the study period.
Labcharoenwongs	2012	0.1%	ointment	VKC	2 times a day for 2 months	Signs and symptoms improvement after one month of treatment.
Fukushima	2014	0.1%	Lacrimart	VKC, AKC	2 times a day for 6 months	Signs and symptoms improvement after one month of treatment.
Pucci	2015	0.1%	Lacrimart	VKC	3 times a day for 3 weeks	Signs and symptoms improvement after three weeks of treatment.
Al-Amri	2016	0.1%	ointment	VKC	Variable	Signs and symptoms improvement after six weeks of treatment.
Chatterjee	2016	0.03%	ointment	VKC	3 times a day for 3 months	Signs and symptoms improvement after four weeks of treatment.
Barot	2016	0.1%	ointment	VKC, AKC	2 times a day for 3 months	Signs and symptoms improvement after four weeks of treatment.

Miyazaki[2]	2017	0.1%	Lacrimart	VKC, AKC	2 times a day for 3 months	Signs improvement after three months of treatment.
**Compositional/stability studies**						
Zeng	2016	0.1%	Niosomes and mucoadhesive hyaluronic acid	rabbits	-	The hybrid system facilitated FK506 ocular delivery on mucoadhesion, precorneal retention, aqueous humor pharmacokinetics and transcorneal permeability
Ezquer-Garin	2017	0.03%	Lacrimart	In vitro	-	Tacrolimus in eye drop solution was stable for 20 days when stored at 25 °C and for at least 85 days when stored at 2–8 °C or between −15 and −25 °C
Luaces-Rodríguez	2018	0.03%	BBS (Balanced Salt Solution), PVA (Polyvinyl alcohol) and Hyaluronic Acid	Rats, VKC, AKC	6 months	Formulations with PVA and Hyaluronic Acid present more retention time on the ocular surface of rats than PBS

© 2020 by the authors. Licensee MDPI, Basel, Switzerland. This article is an open access article distributed under the terms and conditions of the Creative Commons Attribution (CC BY) license (http://creativecommons.org/licenses/by/4.0/).
